# Portable Infrared Laser Spectroscopy for On-site Mycotoxin Analysis

**DOI:** 10.1038/srep44028

**Published:** 2017-03-09

**Authors:** Markus Sieger, Gregor Kos, Michael Sulyok, Matthias Godejohann, Rudolf Krska, Boris Mizaikoff

**Affiliations:** 1 Ulm University, Institute of Analytical and Bioanalytical Chemistry, Albert-Einstein-Allee 11, 89081 Ulm, Germany; 2 McGill University, Atmospheric and Oceanic Sciences, 805 Sherbrooke Street West, Montreal, QC, H3A 0B9, Montreal, Canada; 3 University of Natural Resources and Applied Life Sciences, IFA-Tulln, Konrad Lorenz Straße 20, A-3430 Tulln, Austria; 4 MG Optical Solutions GmbH, Industriestr. 23, 86919 Utting/Ammersee, Germany

## Abstract

Mycotoxins are toxic secondary metabolites of fungi that spoil food, and severely impact human health (e.g., causing cancer). Therefore, the rapid determination of mycotoxin contamination including deoxynivalenol and aflatoxin B_1_ in food and feed samples is of prime interest for commodity importers and processors. While chromatography-based techniques are well established in laboratory environments, only very few (i.e., mostly immunochemical) techniques exist enabling direct on-site analysis for traders and manufacturers. In this study, we present MYCOSPEC - an innovative approach for spectroscopic mycotoxin contamination analysis at EU regulatory limits for the first time utilizing mid-infrared tunable quantum cascade laser (QCL) spectroscopy. This analysis technique facilitates on-site mycotoxin analysis by combining QCL technology with GaAs/AlGaAs thin-film waveguides. Multivariate data mining strategies (i.e., principal component analysis) enabled the classification of deoxynivalenol-contaminated maize and wheat samples, and of aflatoxin B_1_ affected peanuts at EU regulatory limits of 1250 μg kg^−1^ and 8 μg kg^−1^, respectively.

Mycotoxins are toxic secondary metabolites of fungal species that infest agricultural commodities such as maize, wheat, and peanuts. Aflatoxins (AF) in peanuts and deoxynivalenol (DON) in cereals are among the most relevant toxic fungal metabolites, due to their global occurrence, danger to human and animal health, and resulting financial damage^1^,^2^,^3^,^4^. Aflatoxin B_1_ is known as a human carcinogen, while DON - as well as aflatoxin B_1_ - may exert immunosuppressive effects^5^,^6^. Therefore, rapid testing and classification routines during import, storage, and processing of food and feed relevant commodities are increasingly demanded. Nowadays, chromatographic methods, e.g., liquid chromatography combined with mass spectrometry (LC-MS) are mainly used due to their high sensitivity, selectivity, and comprehensive validation^7^,^8^,^9^. While highly suited for regulatory testing, advanced laboratory facilities along with highly qualified personnel are required, thus rendering these methods of limited practicality and costly for rapid on-site analysis by importers, traders, and food and feed companies. Consequently, despite the availability of several commercial enzyme-linked immune-sorbent assays (ELISA) there is still a lack of rapid screening methods, which require minimal sample preparation and can be used on site^10^,^11^.

Analytical methods utilizing mid-infrared (MIR; 4000–400 cm^−1^, 2.5–20 μm) spectroscopy are nowadays routinely used for the detection and identification of molecular constituents due to the inherent discriminatory information provided by the resulting vibrational, vibro-rotational, and rotational signatures^12^. In particular, infrared attenuated total reflection (IR-ATR) spectroscopy has emerged as a viable strategy for rapid analysis for mycotoxin-contaminated commodities, as reported for maize, wheat, raisins, and peanuts^13^. While infrared spectra provide inherent molecular information, the evaluation of individual absorption bands is of limited utility, as IR spectroscopic techniques usually rely on the detection of matrix changes due to physical alterations caused by fungal contaminations (i.e., content of fatty acids, proteins) rather than detection of individual metabolites^14^. Furthermore, changes in the spectrum of commodities are minute, and are partially superimposed by features of healthy matrix components^15^,^16^,^17^,^18^. Therefore, multivariate data evaluation and classification strategies (i.e., chemometrics) are usually required taking advantage of selected spectral windows associated with changes related to fungal contamination for establishing appropriate classification and quantification schemes. MIR spectral information obtained during routine food and feed quality assessment is most commonly evaluated via principle component analysis (PCA)^19^,^20^,^21^.

Recently emerging analytical measurement strategies have demonstrated the utility of quantum cascade lasers (QCLs) in combination with thin-film waveguides as a promising alternative to commonly used Fourier transform infrared (FTIR) spectroscopy techniques^22^,^23^,^24^. Due to their exceedingly compact dimensions, high output power, operational stability, and broad tunability (>400 cm^−1^ per device), QCLs are unambiguously accepted as the most advanced light source facilitating compact and portable MIR spectroscopy and sensing schemes^13^,^25^. Thin-film waveguides with a thickness on the order of or below the propagating wavelength ideally complement these light sources ensuring single mode light propagation behavior. Hence, an evanescent field extending from the surface of appropriate high-refractive index waveguide materials into the adjacent sample appears continuous and intense along the entire waveguide structure, as an increased fraction of the propagating mode is forced towards the waveguide-sample interface as compared to conventional macroscopic infrared attenuated total reflection (IR-ATR) waveguides and crystals^26^,^27^. Quantitative evanescent field absorption (A) utilizing thin-film waveguides may accordingly be described via a pseudo-Lambert-Beer relationship A = (εcl)r, where ε is the molar absorptivity, c is the concentration of the analyte, l is the equivalent optical path length, and r is the fraction of radiation power residing outside the waveguide core (i.e., within the evanescent field). Consequently, any intensity enhancement of the evanescent field above the waveguide surface directly increases the obtainable signal-to-noise ratio (SNR), and thus, the overall sensitivity of absorption measurements using such thin-film waveguides^24^,^28^. Consequently, the high spectral density provided by QCLs in combination with the sensitive thin-film waveguides appear ideal for the spectroscopic investigation of minor components and minute spectral changes in complex matrices^29^.

In this study, we demonstrate an innovative approach for direct spectroscopic mycotoxin contamination analysis at EU regulatory limits for the first time utilizing QCL-based spectroscopy in combination with GaAs/AlGaAs thin-film waveguides. An extraction and measurement procedure for maize, wheat, and peanuts was developed providing pronounced spectral features within the tuning range of a single QCL laser light source yielding information on alterations of the sample matrix caused by fungal infection, which in turn enables inferring actual mycotoxin contamination. This strategy enabled the classification of contaminated and uncontaminated samples for different commodities at EU regulatory limits.

## Results

### MYCOSPEC prototype

**Figure 1:**
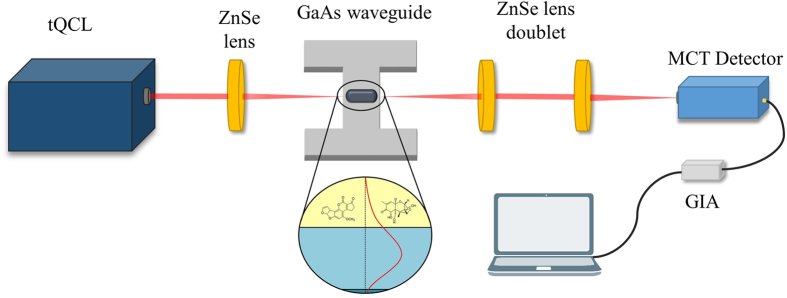
Schematic of the MYCOSPEC sensor system comprising a tunable quantum cascade laser, ZnSe lenses, a GaAs/AlGaAs thin-film waveguide slab, and a TE-cooled MCT detector.

A mid-infrared sensor system based on GaAs/AlGaAs thin-film waveguides in combination with a broadly tunable quantum cascade laser (tQCL) was designed for direct mycotoxin analysis in foodstuff. The experimental scheme illustrated in Fig. 1 comprises a tQCL operated in pulsed mode providing MIR radiation with a tuning range of ~315 cm^−1^ at a repetition rate at 100 kHz and a laser line width <1 cm^−1^. MIR radiation is coupled into a GaAs thin-film waveguide serving as the optical transducer for evanescent field absorption measurements aligned within a stainless steel waveguide assembly. The waveguide structure was optimized for the spectral range of interest (i.e., 1820–1560 cm^−1^) via finite element method (FEM) simulations ensuring single mode behavior in z-direction^30^. Excellent optical throughput and maximized evanescent field intensity were obtained via a 6 μm thin GaAs waveguide structures deposited onto a 6 μm Al_0.2_Ga_0.8_As optical buffer layer residing at the surface of a GaAs wafer substrate. A thermoelectrically cooled mercury-cadmium-telluride (MCT) detector and a gated integrated amplifier (GIA) complemented the optical setup.

### Infrared spectroscopy of foodstuff extracts for mycotoxin analysis

**Figure 2:**
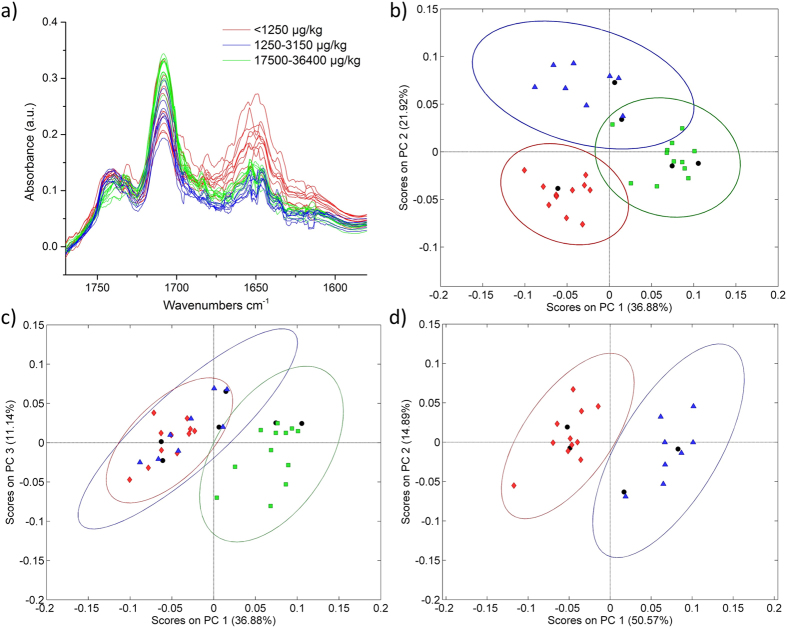
(**a**) QCL spectra (single wavelength sweeps) of 24 maize extracts with different DON contamination levels. PCA score plots of QCL spectra (1770–1580 cm^−1^) of maize with various DON contamination ranges (<1250 μg kg^−1^, red; 1250–3150 μg kg^−1^, blue; 17500–36400 μg kg^−1^, green). (**b**) PC1 vs PC2, and (**c**) PC1 vs PC3 of the entire dataset. (**d**) PCA of a reduced dataset, where samples with contaminations well above the EU regulatory limits were excluded. Validation samples are indicated as black dots.

Herein, the capability of MYCOSPEC for the detection of mycotoxins in foodstuff was demonstrated by investigating maize, wheat, and peanut extracts. Alterations of the sample matrix caused by fungal infection were quantified via differences in their IR spectra. A decrease of the amide I band at around 1655 cm^−1^, and changes in the C=O stretching bands at 1710 cm^−1^ and 1740 cm^−1^ were observed, which may be attributed to fatty acids and esters^31^,^32^, respectively, as shown in Fig. 2a for maize extracts. While these changes are visually evident at high mycotoxin concentrations, only minor changes are recognizable for low contamination levels requiring multivariate data mining strategies. In contrast to previously described MIR methods where ground samples were analyzed^16^,^17^, extracts were used herein providing more intense and pronounced spectral features correlated to fungal damage and mycotoxin contamination levels. Furthermore, by selecting appropriate solvents the intensity of the most characteristic absorption features may be optimized, while the contribution of unspecific bands may accordingly be reduced while simultaneously enhancing the discrimination during multivariate classification. Solvents were selected featuring high vapor pressure for rapid analysis, and the absence of absorption bands within the spectral range of interest avoiding any interference of sample and solvent absorptions. For cereals, methanol was identified as the most suitable solvent due to the ability to extract fatty acids and esters, as well as proteins. As proteins in peanuts are insoluble in most organic solvents (e.g., acetonitrile, methanol, ethanol, etc.), ethanol was used to maximize the amount of extracted fatty acids and esters^33^.

### Analysis of DON-contaminated maize samples

A dataset of 24 samples comprising eight maize samples with DON concentrations below the EU regulatory limit for unprocessed maize (<1250 μg kg^−1^), eight samples just above the limit (1250–3150 μg kg^−1^), and eight highly contaminated samples (17500–36400 μg kg^−1^) was recorded. All samples were previously analyzed via validated LC-MS/MS methods^8^. For the analysis, 10 μL of the solvent extract were applied onto the waveguide surface and allowed to evaporate prior to spectral recording. Importantly, a spectrum obtained from a single scan (i.e., tuning sweep across the wavelength band provided by the tQCL) was sufficient for obtaining useful sample spectra calculating the absorbance following A = −log(I/I_0_). The noise observed within the spectra is mainly attributed to minute changes in moisture and small laser fluctuations. To examine differences between the three contamination levels, a PCA-based data evaluation strategy was implemented providing statistically significant separation of the data clusters associated with the three contamination levels along principal components (PC) 1 and 2, without any data pre-treatment (Fig. 2b). Along PC1, the separation was mainly attributed to changes in the shoulders of the C=O stretching band at 1710 cm^−1^, while PC2 predominantly captured differences of the main C=O absorption and differences in the amide vibrations around 1655 cm^−1^. PC3 apparently separated the spectra due to changes in the ester band at 1740 cm^−1^. Along the PC3 axis, samples at EU limits clustered with samples below the limit, however, remain separated from highly contaminated samples, as shown in Fig. 2c. This behavior can be explained due to the wide concentration range of DON contaminations, as the damage particularly evident via the C=O band at 1740 cm^−1^ caused from the fungal infestation increased strongly at elevated DON contaminations. Consequently, the combination of the information provided by PC2 and PC3 led to a first decision whether a sample was highly contaminated or below and slightly above the EU regulatory limit, respectively. Thus, if the PCA indicates a low contamination, a second PCA was be performed considering only contaminations around the regulatory limits. In Fig. 2d, the separation of samples below and just above the EU limits is depicted. A statistically significant separation between both contamination levels was clearly evident along PC1 and PC2. While the information content of PC1 was similar to the PCA performed for all three contaminations, PC2 now also included changes in the ester band at 1740 cm^−1^. After establishing the calibration model, a validation was performed with two samples of each contamination level (black dots in Fig. 2b–d). As illustrated in Fig. 2, all six validation samples were correctly assigned to the appropriate contamination level, i.e., data clusters.

### Analysis of DON-contaminated wheat samples

**Figure 3:**
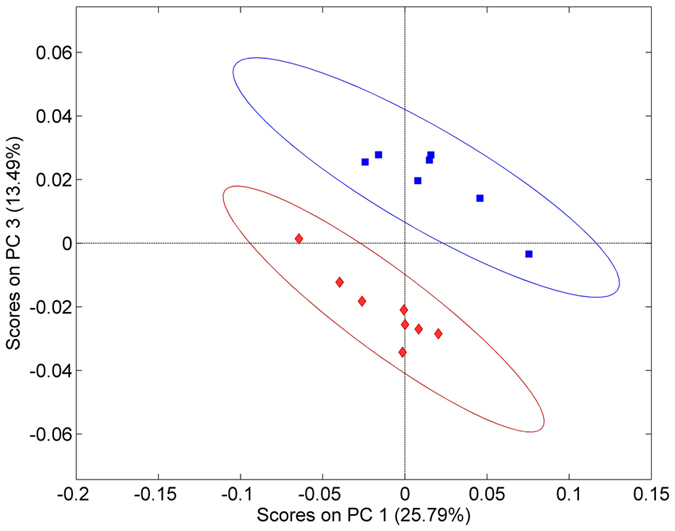
PCA score plot of wheat samples separated in two DON contamination ranges (>1250 μg kg^−1^, red; 1250–2700 μg kg^−1^, blue).

Although the chemical composition of wheat is similar to maize, the fraction of fatty acids and esters is lower^34^, which usually results in a reduced separation of the spectral data sets during PCA analysis. Due to the enhancement of specific spectral bands during the extraction process and the exquisite sensitivity of thin-film waveguides, a separation at EU limits was achieved for the first time using MIR spectroscopic sensing techniques (Fig. 3). Along PC1, variances are mainly attributed to changes in the amide region ~1650 cm^−1^ and the C=O band (i.e., esters) at 1740 cm^−1^, while PC3 considers changes in the C=O stretching band at 1710 cm^−1^. Variances in PC2 are mainly attributed to rather unspecific changes and do not provide for appropriate clustering of the data.

### Analysis of AFB_1_ in Peanuts samples

**Figure 4:**
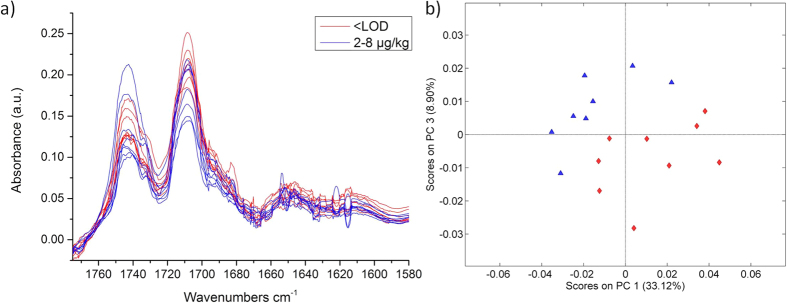
(**a**) QCL spectra of 17 peanut extracts with different aflatoxin B_1_ concentrations. (**b**) PCA score plots of QCL spectra (1770–1680 cm^−1^) of peanuts separated in two aflatoxin B_1_ contamination ranges (<LOD, red; 2–8 μg kg^−1^, blue).

As the proteins in peanuts are insoluble in methanol, the extraction process was adapted and ethanol was used to maximize the amount fatty acids in the extraction solution. The spectral window used during PCA analysis was limited to 1760–1670 cm^−1^ for reducing the effects of unspecific bands, i.e., the C=C stretching vibration at 1650 cm^−1^, as shown in Fig. 4a. A score plot is shown in Fig. 4b for nine uncontaminated and eight contaminated samples (2–8 μg kg^−1^ Aflatoxin B_1_), respectively. Along PC1, variances are mainly attributed to changes in the ester band at 1744 cm^−1^, while variances in PC3 are associated with the C=O stretching band at 1710 cm^−1^. Although the separation is less pronounced than for cereals, a separation of aflatoxin B_1_ contaminated peanuts at EU regulatory limits has been achieved for the first time using infrared spectroscopic sensing techniques.

## Discussion

A mid-infrared spectroscopic sensing method using tunable quantum cascade lasers and GaAs thin-film waveguides was developed providing the required sensitivity and spectral resolution for enabling rapid on-site determination of mycotoxin contaminations in various agricultural commodities at EU regulatory concentration levels. An extraction procedure with minimal sample preparation and solvent consumption facilitates sufficiently pronounced spectral differences of the dominant characteristic components related to fungal contamination and apparently affected during mycotoxin production. Classification of the contamination levels at EU regulatory limits were demonstrated for deoxynivalenol-contaminated wheat and maize, and for aflatoxin B_1_ contaminated peanuts. The laser-based spectroscopic sensing system provides rapid data acquisition, automated data analysis and classification, and an inherent potential for further miniaturization rendering this analytical concept highly suitable for in-field use, e.g., for at-point testing during commodity import, storage, and processing. Hence, MYCOSPEC may indeed serve as an on-site decision-making tool for commodity importers and manufacturers prior to conventional laboratory-based analyses finally demanded by a food safety authority. Given the global increase in mycotoxin contaminations due to climate change and extreme weather conditions, it is apparent that rapid, reliable, and portable mycotoxin detection methods at moderate cost are in demand for ensuring food and feed safety.

## Methods

### Sample preparation

Maize and wheat samples were cultivated at the University of Life Sciences and Natural Resources (BOKU), and either naturally or artificially infected. For maize, DON concentrations just above the EU regulatory limits were achieved via toothpick inoculation with *Fusarium verticillioides*; higher concentrations were obtained via injection of *F. graminearum* into the silk channel. Wheat samples were naturally infected and inoculated with *F. graminearum*. Peanut samples were collected from public markets in different regions of Tanzania. All samples were milled to a particle size <1 mm, and stored at +4 °C.

### Extraction procedure

Samples were extracted from ground cereals and peanuts by shaking with methanol for wheat and maize, and with ethanol for peanuts, respectively. 200 μg of ground sample was mixed with 800 μL of solvent. After centrifugation, the solvent was transferred into a new vial and stored in the refrigerator until further analysis.

### MYCOSPEC sensor system

Thin-film GaAs/AlGaAs waveguide structures were designed and optimized for the spectral regime of interest, i.e., 1820–1560 cm^−1^ via finite element simulations (COMSOL Multiphysics 4.4, COMSOL, Burlington, MA, USA). A 6 μm GaAs waveguide on top of a 6 μm Al_0.2_Ga_0.8_As optical buffer layer (at a GaAs wafer substrate) with final chip dimensions of 10 × 5 mm was mounted in a stainless steel waveguide alignment assembly developed at the Institute of Analytical and Bioanalytical Chemistry, Ulm University. As MIR light source, a MIRcat QCL spectrometer (Daylight Solutions Inc., San Diego, CA, USA.) comprising a single tunable QCL module was used covering the spectral range of 1820–1560 cm^−1^. The QCL was operated in pulsed mode with a repetition rate of 100 kHz, and a pulse width of 100 ns. For signal detection, a three-stage thermoelectrically cooled photoconductive mercury cadmium telluride detector (PCI-3TE-MCT, Vigo, Ożarów Mazowiecki, Poland) was used. Spectra acquisition was performed via a gated-integrating amplifier (GIA) converting the energy of each laser pulses into a continuous signal, which was then recorded via a LabView script (LabView 2014, SP1, V 14.0.1f3, National Instruments GmbH, Munich, Germany). The laser was operated in internal sweep mode with a scan speed of 2 cm^−1^/s, while data points were recorded every 200 ms resulting in a theoretical spectral resolution of 0.4 cm^−1^.

### Spectral data processing

Absorption spectra were calculated in a spreadsheet program (OriginPro 9 G, OriginLab Corporation, Northampton, U.S.A.) following A = −log(I/I_0_) using single background and sample scans, respectively. An adjacent-averaging smoothing with a 10 points window was applied to the calculated absorption spectra for reducing influences of rotational bands of water vapor during principle component analysis. The PLS Toolbox 7.95 (Eigenvector Research, Inc., Manson, Washington, U.S.A.) for MATLAB (Version 2012b) was used for PCA analysis.

## Additional Information

**How to cite this article:** Sieger, M. *et al*. Portable Infrared Laser Spectroscopy for On-site Mycotoxin Analysis. *Sci. Rep.* **7**, 44028; doi: 10.1038/srep44028 (2017).

**Publisher's note:** Springer Nature remains neutral with regard to jurisdictional claims in published maps and institutional affiliations.
